# The quantitative proteomics analysis of multi-stage gastric mucosal lesions associated with *Helicobacter pylori* infection

**DOI:** 10.1371/journal.pone.0353347

**Published:** 2026-07-31

**Authors:** Xiaoxi Huang, Jiamei Ma, Yening Xiao, Jing Pan, Qin Xu, Xifan Cao, Yongchao Liu, Xiang Yi, Fang Tian

**Affiliations:** The First Affiliated Hospital of Hainan University, Haikou People’s Hospital, Haikou, Hainan, China; Jamia Millia Islamia Central University: Jamia Millia Islamia, INDIA

## Abstract

**Background:**

*Helicobacter pylori* (*H. pylori*) infection is the principal etiological factor for gastric cancer (GC), which ranks as the fourth leading cause of cancer-related mortality globally. Although the Correa cascade describes the stepwise progression from chronic gastritis to intestinal-type GC, the molecular events underlying this “inflammation-to-cancer” transition remain incompletely characterized.

**Objective:**

This study aimed to delineate stage-specific proteomic alterations across *H. pylori*-associated gastric mucosal lesions and to identify candidate progression-associated biomarkers in GC.

**Methods:**

Label-free quantitative proteomic analysis (LC-MS/MS) was performed on gastric tissue specimens from 22 patients stratified into four histopathological stages: *H. pylori*-negative chronic non-atrophic gastritis (HpN-CG), *H. pylori*-positive chronic non-atrophic gastritis (HpC-CG), *H. pylori*-positive chronic atrophic gastritis (HpC-CAG), and intestinal-type GC. Functional enrichment (GO, KEGG), temporal clustering (Mfuzz), and protein-protein interaction (PPI) network analyses were conducted. Key hub genes were externally assessed using GEPIA RNA-seq data from TCGA and GTEx.

**Results:**

A total of 6,019 proteins were quantified. Differential expression analysis identified 771 DEPs between HpC-CG and HpN-CG, 101 DEPs between HpC-CAG and HpC-CG, and 535 DEPs between GC and HpC-CAG. Immune response pathways were up-regulated upon *H. pylori* infection, whereas oxidative phosphorylation was progressively suppressed throughout disease progression. Mfuzz clustering revealed that Cluster 4 ribosome-biogenesis proteins, including NIP7 and PDCD11 identified in the proteomic/PPI analysis, exhibited continuous up-regulation from precancerous stages to GC. External RNA-expression validation supported significant up-regulation of seven Cluster 4 hub genes, whereas Cluster 6 proteins did not show concordant transcript-level down-regulation.

**Conclusion:**

This study provides a comprehensive proteomic landscape of *H. pylori*-driven gastric carcinogenesis. Cluster 4 ribosome-biogenesis proteins represent candidate progression-associated protein markers requiring further protein-level validation, while the progressive decline in oxidative phosphorylation underscores mitochondrial dysfunction as a hallmark of disease progression.

## Introduction

*H. pylori* is a recognized pathogen and class I carcinogen [1]. Although infection rates in China have declined from 58.3% to approximately 40% in recent decades, likely due to socioeconomic and healthcare improvements, the substantial population base means *H. pylori* infection remains a significant public health burden [2]. Notably, only 1–3% of infected individuals develop gastric cancer (GC) [3]. Once established, *H. pylori* infection persists without spontaneous eradication and can drive a continuum of gastric mucosal inflammation leading to cancer.

Globally, GC ranks fifth in incidence and fourth in cancer-related mortality [1]. In China, it is the second most common cancer and the second leading cause of cancer death, imposing substantial health and economic burdens [4,5]. According to the Lauren classification, GC comprises intestinal and diffuse types, with the intestinal type generally having a more favorable prognosis [6]. The Correa cascade model, proposed in 1992, describes the progression of intestinal-type GC initiated by *H. pylori* infection. This model outlines sequential pathological stages: superficial gastritis, chronic non-atrophic gastritis (CG), chronic atrophic gastritis (CAG), intestinal metaplasia (IM), dysplasia (DYS), and invasive adenocarcinoma [7]. Prospective studies confirm that long-term *H. pylori* infection promotes the development of these precancerous lesions [8,9]. However, while the Correa hypothesis frames CG, CAG, IM, DYS, and GC as a continuum, it does not quantify the progression risk at each stage, and the molecular mechanisms driving this “inflammation-to-cancer” transition remain poorly understood.

Proteomics, the large-scale analysis of proteins, offers a powerful approach to identify novel biomarkers and elucidate disease mechanisms [10,11]. While proteomic studies in GC have identified numerous proteins associated with invasion, metastasis, tumorigenesis, and prognosis [12–14], few have systematically examined the proteomic changes across the series of precancerous lesions preceding intestinal GC, particularly in the context of concurrent *H. pylori* infection and potential diffuse GC components. Applying proteomic technology to profile gastric mucosal tissues at distinct histological stages associated with *H. pylori* infection is therefore crucial. This approach can pinpoint key proteins and molecular events in *H. pylori*-induced gastric lesion development, unravel early biological events in GC pathogenesis, and provide a foundation for exploring *H. pylori* virulence mechanisms and developing diagnostic and individualized therapeutic strategies for GC.

## Materials and methods

### Study population and sample collection

This single-center, cross-sectional study was conducted at Haikou People’s Hospital between March and December 2023. The study protocol was approved by the Institutional Ethics Committee of Haikou People’s Hospital (Approval No. 2023−065) in accordance with the Declaration of Helsinki. All participants provided written informed consent. Adult patients aged 18–80 years were enrolled based on the following inclusion criteria: (1) endoscopic evidence of gastric mucosal lesions (single erosion, masses, ulcers, atrophy, intestinal metaplasia, or suspected malignancy) with histopathological confirmation of chronic non-atrophic gastritis, chronic atrophic gastritis, or intestinal-type GC; (2) *H. pylori* infection status determined by ^13^C-urea breath test (UBT) combined with clinical history; and (3) provision of written informed consent.

Exclusion criteria comprised: (1) prior *H. pylori* eradication therapy; (2) history of gastric surgery or anticancer treatment (chemotherapy, radiotherapy, or targeted therapy) of severe chronic diseases or other malignancies; (3) concurrent severe systemic diseases or other malignancies; (4) heavy smoking (>10 cigarettes/day), excessive alcohol consumption (>30 g ethanol/day), or obesity (BMI ≥ 30 kg/m^2^); and (5) diffuse-type or mixed-type GC according to the Lauren classification.

### Histopathological evaluation

Diagnoses were established through integrated endoscopic and histopathological assessment. Five biopsy specimens were obtained from the most severely affected region of the gastric antrum in each patient. One specimen was immediately fixed in 10% neutral-buffered formalin, paraffin-embedded, and sectioned at 5 μm thickness. Sections were stained with hematoxylin and eosin (H&E) for morphological evaluation and with Giemsa stain for *H. pylori* detection. Histopathological parameters assessed included: chronic inflammatory infiltration, glandular atrophy, intestinal metaplasia, and *H. pylori* colonization density. GC cases were classified according to the Lauren criteria. All specimens were independently reviewed by a board-certified gastrointestinal pathologist blinded to clinical data.

The remaining four biopsy specimens were snap-frozen in liquid nitrogen within 30 minutes of collection and stored at −80°C until proteomic analysis. For surgical GC specimens, tumor tissue (0.5 × 0.5 cm) was dissected from the central region within 30 minutes of resection, rinsed with ice-cold sterile saline, flash-frozen in liquid nitrogen, and maintained at −80°C.

### Protein extraction

Frozen tissue specimens were pulverized in liquid nitrogen-chilled mortars. Tissue powder was lysed in buffer containing 1% SDS (Amresco), 1% protease inhibitor cocktail (Merck Millipore), 3 μM trichostatin A (MedChemExpress), and 50 mM nicotinamide (Sigma-Aldrich). Following precipitation with 10% trichloroacetic acid/acetone (Sigma-Aldrich), samples underwent pulsed ultrasonication (35% amplitude, 3 s on/5 s off, 3 min total duration). Lysates were centrifuged at 18,000 × g for 10 min at 4°C, and supernatants were collected. Protein concentrations were determined using a bicinchoninic acid (BCA) assay kit (Beyotime).

### Trypsin digestion

Protein aliquots (200 μg per sample) were precipitated with ice-cold acetone (1:4, v/v) at −20°C for 2 h. Pellets were washed with acetone, resuspended in 200 mM tetraethylammonium bromide (TEAB; Sigma-Aldrich), and sonicated for 5 min. Reduction was performed with 5 mM dithiothreitol (DTT) at 56°C for 30 min, followed by alkylation with 11 mM iodoacetamide (IAA) at room temperature for 15 min in dark. Proteins were digested with sequencing-grade trypsin (1:50, w/w; Promega) at 37°C overnight. Peptides were acidified to pH 2–3 with 10% trifluoroacetic acid (TFA; Sigma-Aldrich), centrifuged at 18,000 × g for 10 min, and desalted using C18 solid-phase extraction cartridges (Thermo Fisher Scientific). Peptides were eluted with 80% acetonitrile/0.1% TFA and quantified by BCA assay.

### LC-MS/MS analysis

Peptides were dissolved in mobile phase A (2% acetonitrile, 0.1% formic acid in water) and separated using a NanoElute ultra-high-performance liquid chromatography system (Bruker, Karlsruhe, Germany). Mobile phase B consisted of 100% acetonitrile with 0.1% formic acid. The chromatographic gradient was as follows: 0–70 min, 6–24% B; 70–84 min, 24–35% B; 84–87 min, 35–80% B; 87–90 min, 80% B. The flow rate was maintained at 450 nL/min.

Separated peptides were analyzed on a trapped ion mobility spectrometry-time of flight (TIMS-TOF) Pro mass spectrometer (Bruker, Billerica, USA) equipped with a CaptiveSpray nano-electrospray ion source. The capillary voltage was set at 1.8 kV. Data acquisition was performed in parallel accumulation-serial fragmentation (PASEF) mode with a scan range of m/z 100–1,700. Ten PASEF MS/MS scans were acquired per cycle for precursor ions with charge states 2–5. Dynamic exclusion was set at 30 s to minimize redundant fragmentation. The mass spectrometry proteomics data have been deposited to the ProteomeXchange Consortium via the iProX partner repository [15, 16] with the dataset identifier PXD053524.

### Proteomic data processing

Raw data were analyzed by Maxquant software (version 1.6.15.0). (1) The human UniProt/Swiss-Prot database (Homo_sapiens_9606_SP_20230103.fasta; 20,389 sequence) was used for protein identification. A decoy database and common contaminant sequences were included for false discovery rate (FDR) estimation. FDR thresholds for peptide-spectrum matches and protein identification were set at 1%. (2) Trypsin/P was specified as the proteolytic enzyme, allowing up to two missed cleavages. Minimum peptide length was set at seven amino acid residues. A maximum of five variable modifications per peptide was permitted. First search and main search mass tolerances for precursor ions were 20 ppm and 20 ppm, respectively. Fragment ion mass tolerance was 20 ppm. (3) In the database search engine, cysteine alkylation was set as a fixed modification. Oxidation of methionine and N-terminal acetylation were specified as variable modifications. Proteins with |log_2_ fold change| > 0.585 (corresponding to fold change > 1.5) and p < 0.05 (Student’s t-test) were considered differentially expressed.

### Functional enrichment analysis

Gene Ontology (GO) annotation was performed to classify differentially expressed proteins (DEPs) into biological process (BP), cellular component (CC), and molecular function (MF) categories. Kyoto Encyclopedia of Genes and Genomes (KEGG) pathway analysis was conducted to identify enriched signaling pathways. Enrichment analyses were performed using the clusterProfiler package in R, with visualization using ggplot2. Fisher’s exact test was applied for enrichment analysis against the background of all quantified proteins. Pathways with p < 0.05 were considered significantly enriched.

### Temporal clustering analysis

To characterize dynamic protein expression patterns across disease stages, Mfuzz soft clustering was performed. Protein abundance values were log2 logarithmic conversion and standardized. Proteins with standard deviation > 0.6 across groups were retained for clustering analysis. The Mfuzz package in R was employed with the following parameters: fuzzification coefficient (m) = 2; number of clusters (k) = 6.

### Protein-protein interaction (PPI) network analysis

DEPs from selected clusters were imported into STRING database (version 11.5) to construct PPI networks under moderate confidence settings (interaction score > 0.4). The resulting networks were visualized and analyzed in Cytoscape (version 10.0). Hub proteins were identified using the CytoHubba plugin with the maximal clique centrality (MCC) algorithm, extracting the top 10 hub genes with highest network connectivity.

### Validation of hub gene expression

Expression levels of the identified hub genes in GC versus normal gastric tissue were externally assessed using the Gene Expression Profiling Interactive Analysis (GEPIA) database (http://gepia.cancer-pku.cn/), which integrates RNA-seq data from The Cancer Genome Atlas (TCGA) and Genotype-Tissue Expression (GTEx) projects.

### Statistical analysis

Statistical analyses were conducted using SPSS 26.0 and Python 3.7.6. Continuous variables with normal distribution were expressed as mean ± standard deviation (SD) and compared using one-way ANOVA. Non-normally distributed variables were presented as median (interquartile range, IQR) and analyzed using Kruskal-Wallis test. Categorical variables were described by frequency and percentage and compared using Fisher’s exact test or chi-square test as appropriate. For proteomic differential analysis, homogeneity of variance was assessed using Levene’s test. Independent samples t-test was applied when variance homogeneity was satisfied; otherwise, Welch’s t-test was used. Statistical significance was defined as p < 0.05.

## Results

### Patient characteristics

**Fig 1:**
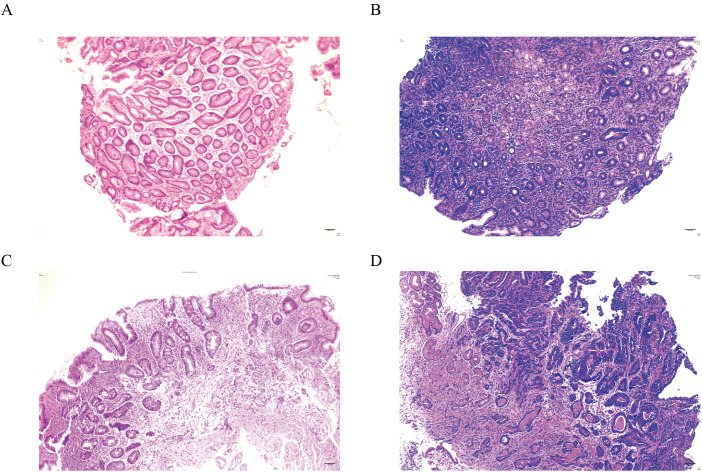
Representative histopathological images of gastric mucosal lesions (H&E staining, × 100). (A) Chronic non-atrophic gastritis with no *H. pylori* infection (HpN-CG); (B) chronic non-atrophic gastritis with current *H. pylori* infection (HpC-CG); (C) chronic atrophic gastritis with current *H. pylori* infection (HpC-CAG); and (D) intestinal type gastric cancer (GC).

Twenty-two patients meeting inclusion criteria were enrolled and categorized into four groups: *H. pylori*-negative chronic non-atrophic gastritis (HpN-CG, n = 6), *H. pylori*-positive chronic non-atrophic gastritis (HpC-CG, n = 6), *H. pylori*-positive chronic atrophic gastritis (HpC-CAG, n = 6), and intestinal-type GC (n = 4). No significant differences were observed among groups in baseline demographic characteristics, including age, sex distribution, smoking status, and alcohol consumption (Table 1). Representative histopathological images of each group are shown in Fig 1.

**Table 1:** Baseline characteristics of study participants.

Group	HpN-CG^1^	HpC-CG^2^	HpC-CAG^3^	GC^¶^	*p*-value
Sample size	6	6	6	4	
Age (years ± SD)	43.17 ± 9.58	55.33 ± 14.35	53.17 ± 9.77	60.50 ± 10.85	0.13
Sex					
Male	4	4	2	2	0.783
Female	2	2	4	2	
Smoke					
No smoking	4	4	5	2	0.92
Smoking	2	2	1	2	
Drink					
No drinking	4	3	6	2	0.252
Drinking	2	3	0	2	

Notes: H. pylori-negative chronic non atrophic gastritis (HpN-CG); ^2^ Chronic non-atrophic gastritis positive for H. pylori (HpC-CG); ^3^ Chronic atrophic gastritis positive for H. pylori (HpC-CAG); ¶ Intestinal type gastric cancer (GC)

### Protein identification and quantification

Label-free quantitative proteomic analysis was performed on gastric mucosal tissue specimens from these 22 patients. MaxQuant processing of raw data yielded 3,636,872 MS/MS spectra, of which 1,133,120 (31.16%) were successfully matched to peptide sequences. In total, 83,182 peptides corresponding to 6,019 proteins were identified, including 80,034 unique peptides.

To address the robustness of protein identification, we reviewed the peptide-level evidence available in S1 Table. NIP7 (Q9Y221), a key Cluster 4 protein, was supported by 32.8% sequence coverage, 3 total peptides, 3 unique peptides, and 3 razor + unique peptides. Other Cluster 4 hub proteins represented in S1 Table were also supported by multiple unique peptides, including DDX27 (17), EBNA1 BP2 (10), MAK16 (6), WDR12 (9), PNO1 (4), and WDR3 (10). These data support the reliability of the Cluster 4 ribosome-biogenesis signature, although independent protein-level validation in larger cohorts remains warranted.

**Fig 2:**
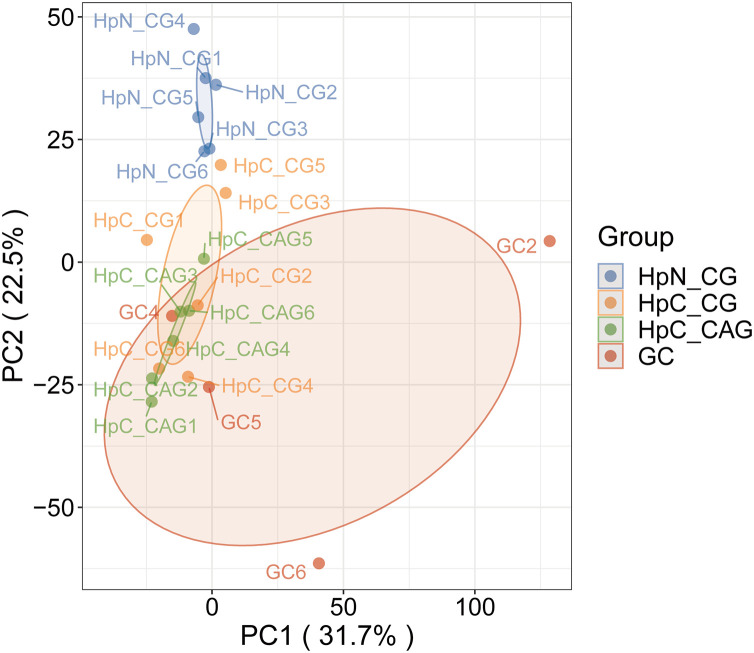
Principal component analysis (PCA) of proteomic profiles. PCA plot based on normalized protein abundance values across 22 samples. Each point represents an individual sample, with colors indicating pathological groups. The horizontal and vertical axes represent PC1 and PC2, respectively.

**Fig 3:**
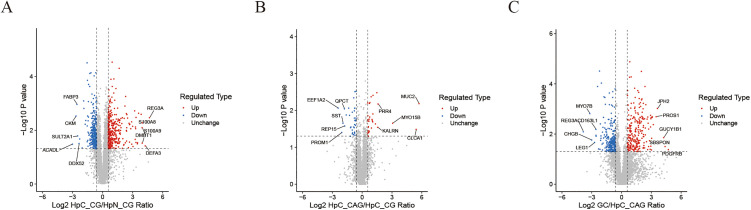
Volcano plots of differentially expressed proteins (DEPs) across comparison groups. (A) HpC-CG vs. HpN-CG groups. (B) HpC-CAG vs. HpC-CG groups. (C) GC vs. HpC-CAG groups. The x-axis represents log2-transformed fold change, and the y-axis represents -log10-transformed p value. Dots indicate differentially expressed proteins. Red and blue dots represent up- and down-regulated proteins, respectively. The top five DEPs in each direction are labeled.

**Fig 4:**
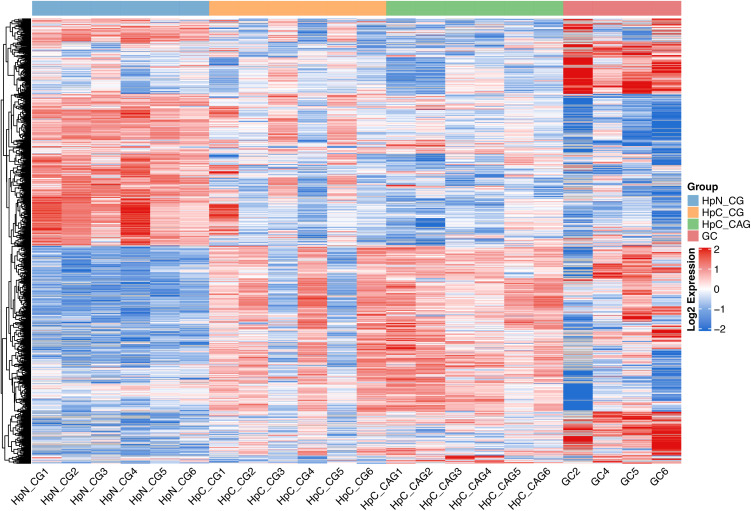
Hierarchical clustering heatmap of differentially expressed proteins (DEPs). Rows represent proteins and columns represent samples. Red means high expression, blue means low expression, and grey means not quantifiable in the corresponding sample.

Principal component analysis (PCA) based on relative quantitative values of all samples was visualized (Fig 2). Comparative analysis showed 771 DEPs between HpC-CG and HpN-CG groups (436 up-regulated and 335 down-regulated). The HpC-CAG vs. HpC-CG comparison revealed 101 DEPs (51 down-regulated and 50 up-regulated), while GC vs. HpC-CAG analysis identified 535 DEPs (274 down-regulated and 261 up-regulated) (Fig 3 and S1 Table). We generated expression heatmaps for the combined DEPs across all comparison groups, requiring protein quantification in at least two-thirds of samples (Fig 4).

### GO enrichment analysis

**Fig 5:**
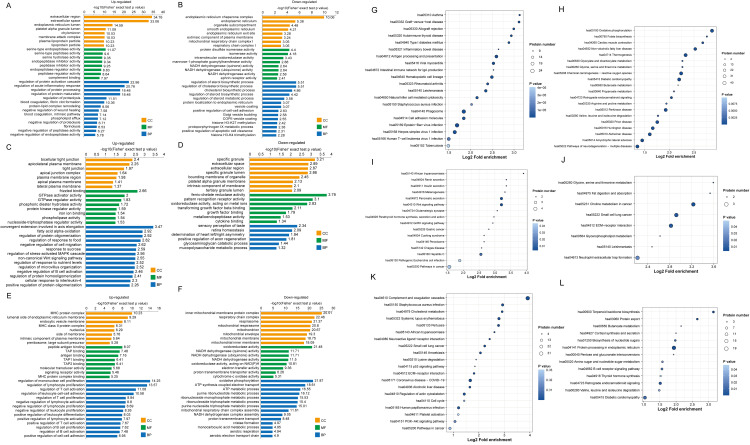
Functional enrichment analysis of differentially expressed proteins (DEPs). (A–F) Gene Ontology (GO) enrichment analysis. (A) Up-regulated and (B) down-regulated DEPs in GC vs. HpC-CAG. (C) Up-regulated and (D) down-regulated DEPs in HpC-CAG vs. HpC-CG. (E) Up-regulated and (F) down-regulated DEPs in HpC-CG vs. HpN-CG. (G–L) KEGG pathway enrichment analysis. (G) Up-regulated and (H) down-regulated DEPs in HpC-CG vs. HpN-CG. (I) Up-regulated and (J) down-regulated DEPs in HpC-CAG vs. HpC-CG. (K) Up-regulated and (L) down-regulated DEPs in GC vs. HpC-CAG. BP, biological process; CC, cellular component; MF, molecular function. Bubble size indicates gene count; color represents adjusted p-value.

For GC vs. HpC-CAG, up-regulated DEPs localized to extracellular regions (p = 1.75e-35) and demonstrated serine-type peptidase activities (p = 8.60e-12 to 4.52e-10), influencing protein activation cascades (p = 2.33e-23) and acute inflammatory responses (p = 1.72e-21). Down-regulated proteins formed endoplasmic reticulum chaperone complexes (p = 8.77e-11) with metabolic enzyme activities, particularly in oxidative phosphorylation and sterol biosynthesis (Fig 5A, 5B).

In the HpC-CAG vs. HpC-CG comparison, up-regulated DEPs participated in cell junction formation (bicellular tight junctions, p = 0.0039; apicolateral plasma membranes, p = 0.0055) and exhibited molecular functions including frizzled binding (p = 0.0021) and GTPase activator activity (p = 0.0099). Biological processes such as convergent extension during axis elongation (p = 0.0003) were identified. Down-regulated DEPs were associated with specific granule formation (p = 0.0006), ferric-chelate reductase activity (p = 0.0001), and pattern recognition receptor activity (p = 0.0008) (Fig 5C, 5D).

GO enrichment analysis of DEPs between HpC-CG and HpN-CG groups revealed distinct functional patterns. Up-regulated proteins were predominantly associated with immune-related processes, including major histocompatibility complex (MHC) protein complex assembly (p = 5.90e-11), antigen processing and presentation (p = 3.33e-8), and leukocyte activation (p = 6.02e-19), particularly involving monocyte, lymphocyte, and leukocyte proliferation and differentiation. Conversely, down-regulated proteins were primarily involved in mitochondrial components and oxidative phosphorylation (p = 1.34e-22) (Fig 5E, 5F).

### KEGG pathway analysis

HpC-CG vs. HpN-CG up-regulated DEPs enriched immune-related pathways including antigen presentation (p = 3.45e-11) and NK cell-mediated cytotoxicity (p = 4.99e-7), while down-regulated DEPs participated in oxidative phosphorylation (p = 5.73e-26) and metabolic pathways. HpC-CAG vs. HpC-CG comparisons revealed Wnt signaling (p = 0.0037) and pancreatic secretion pathways for up-regulated DEPs, with down-regulated DEPs involved in cancer metabolism. GC vs. HpC-CAG showed complement/coagulation cascades (p = 4.45e-38) as the most significant pathway for up-regulated DEPs, while down-regulated DEPs affected endoplasmic reticulum protein processing (p = 2.10e-7) (Fig 5G-5L).

### Temporal clustering of protein expression

**Fig 6:**
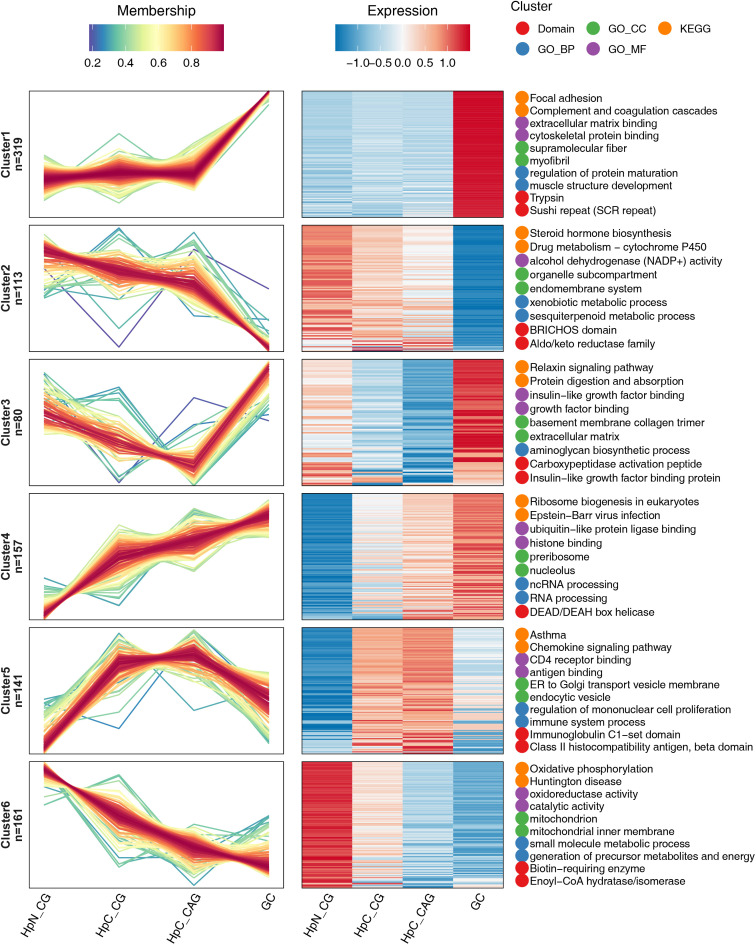
Mfuzz temporal clustering analysis of protein expression patterns. Six clusters identified based on protein expression trajectories across the four pathological stages. Y-axis: standardized expression level; X-axis: disease stage. Color intensity indicates cluster membership value.

Mfuzz soft clustering analysis identified six distinct protein expression patterns across disease progression (Fig 6 and S2 Table). Cluster 1 (319 proteins) displayed a GC-specific up-regulation pattern, with proteins remaining at relatively low levels in HpN-CG, HpC-CG, and HpC-CAG stages but showing marked up-regulation in GC; this cluster was enriched in focal adhesion, ECM-receptor interaction, and PI3K-Akt signaling pathways. Cluster 2 (113 proteins) showed an early up-regulation pattern, with proteins increasing upon *H. pylori* infection (HpC-CG) and remaining elevated thereafter, and was enriched in antigen processing and presentation and phagosome pathways. Cluster 3 (80 proteins) exhibited a biphasic pattern with an initial increase followed by decrease in GC and was enriched in cell adhesion molecules and tight junction pathways. Cluster 4 (157 proteins) showed a progressive up-regulation pattern with continuous increase from HpN-CG through GC and was enriched in ribosome biogenesis in eukaryotes, spliceosome, and RNA transport pathways. Cluster 5 (141 proteins) displayed transient down-regulation in HpC-CAG with recovery in GC and was enriched in protein export and N-glycan biosynthesis pathways. Cluster 6 (161 proteins) exhibited a progressive down-regulation pattern with continuous decrease across disease stages and was enriched in oxidative phosphorylation, thermogenesis, and metabolic pathways.

### PPI network analysis and hub gene identification

**Fig 7:**
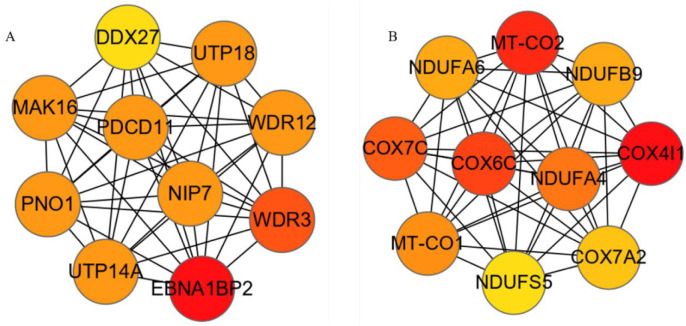
Protein-protein interaction (PPI) network and hub gene identification. (A) PPI network of Cluster 4 proteins with top 10 hub genes identified by MCC algorithm highlighted. (B) PPI network of Cluster 6 proteins with hub genes related to oxidative phosphorylation. Node color intensity indicates MCC score; node size indicates degree.

Given the biological relevance of continuously altered protein expression, we focused on Clusters 4 and 6 for hub gene identification. For Cluster 4, PPI network analysis revealed a highly interconnected module centered on ribosome biogenesis; the top 10 hub genes identified by the MCC algorithm were *NIP7, DDX27, EBNA1 BP2, WDR3, UTP14A, PDCD11, UTP18, MAK16, WDR12*, and *PNO1* (Fig 7A). For Cluster 6, the PPI network identified hub genes predominantly involved in oxidative phosphorylation, including *MT-CO2, NDUFA6, NDUFB9, COX7C, COX4I1, COX6C, NDUFA4, MT-CO1, COX7A2*, and *NDUFS5* (Fig 7B).

### Validation of hub gene expression in external datasets

**Fig 8:**
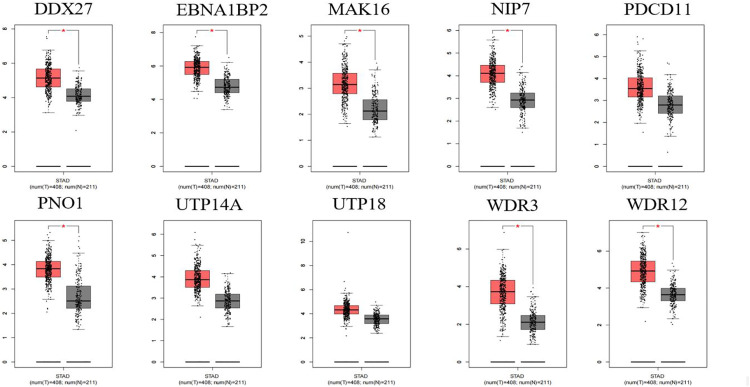
GEPIA RNA-expression validation of Cluster 4 hub genes in gastric cancer. Box plots compare RNA expression levels of the 10 hub genes between TCGA stomach adenocarcinoma (STAD) and GTEx normal gastric tissue. (*p < 0.01).

GEPIA RNA-seq analysis comparing TCGA stomach adenocarcinoma (STAD) samples with GTEx normal gastric tissue revealed that seven of ten Cluster 4 hub genes--*DDX27, EBNA1 BP2, MAK16, NIP7, PNO1, WDR3*, and *WDR12-*-were significantly up-regulated in GC (p < 0.01; Fig 8); *PDCD11, UTP14A*, and *UTP18* did not reach statistical significance at the RNA level. This finding provided external RNA-level support for the proteomic results and the biological relevance of ribosome biogenesis pathway activation in gastric carcinogenesis. For Cluster 6 hub genes, GEPIA RNA-seq analysis revealed no statistically significant differences between GC and normal tissue (S1 Fig), indicating that RNA expression did not substitute for the protein-level decrease observed in Cluster 6.

## Discussion

Gastric carcinogenesis typically follows a well-defined sequence from chronic gastritis to adenocarcinoma, with *H. pylori* infection being the predominant etiological factor [7,17,18]. Our proteomic analysis of gastric mucosa across Correa’s cascade stages revealed distinct molecular signatures associated with disease progression.

The initial *H. pylori* infection phase (HpC-CG vs. HpN-CG) showed up-regulation of immune-related proteins involved in antigen presentation and lymphocyte activation, consistent with established mechanisms of pathogen recognition and inflammatory response initiation [19–21]. These findings align with the paradigm that *H. pylori* infection triggers robust innate and adaptive immune responses through pattern recognition receptors and MHC mediated antigen presentation. This persistent inflammatory microenvironment likely contributes to subsequent malignant transformation through cumulative genetic and epigenetic alterations [22–24].

During progression to atrophic gastritis, we observed significant dysregulation of cell junction proteins and cytoskeletal components, suggesting early structural changes in the gastric epithelium. These findings align with previous reports linking cellular adhesion alterations to pre-neoplastic changes [25].

The most dramatic proteomic changes occurred in the transition to GC, particularly in complement and coagulation pathways. Notably, tissue factor overexpression correlated with disease progression and metastatic potential, supporting its role as a potential biomarker [26,27]. The observed downregulation of mitochondrial respiratory chain components in Cluster 6 proteins further highlights metabolic reprogramming as a key feature of gastric carcinogenesis [28–30]. This observation is consistent with the Warburg effect, wherein cancer cells preferentially utilize glycolysis even in the presence of adequate oxygen. The declining oxidative phosphorylation capacity may represent an adaptive response to hypoxic tumor microenvironments and/or a consequence of mitochondrial DNA mutations accumulated during chronic inflammation.

Our cluster analysis identified the 10 Cluster 4 hub proteins--NIP7, DDX27, EBNA1 BP2, WDR3, UTP14A, PDCD11, UTP18, MAK16, WDR12, and PNO1--as a coordinated ribosome-biogenesis module that increased progressively from HpN-CG through GC. NIP7 is required for accurate pre-rRNA processing and ribosomal subunit maturation [31]. PDCD11, also known as ALG-4/RRP5, participates in early pre-rRNA processing and small-subunit maturation [32,33]. DDX27 is a DEAD-box RNA helicase, whereas EBNA1 BP2, UTP14A, UTP18, MAK16, WDR3, WDR12, and PNO1 are nucleolar or rRNA-processing factors involved in pre-ribosomal particle assembly, small- or large-subunit maturation, and ribosome biogenesis. The shared direction of change among these proteins suggests activation of nucleolar ribosome-production machinery during gastric carcinogenesis rather than isolated dysregulation of a single marker. Because ribosome biogenesis is closely linked to cell growth and malignant proliferation, this Cluster 4 module may reflect increased biosynthetic demand during progression to GC [34].

Our findings have several potential clinical implications. First, the stage-specific proteomic signatures could inform development of multi-marker panels for early GC detection. Second, the coordinated increase of Cluster 4 ribosome-biogenesis proteins may help prioritize candidates for future protein-level validation in larger independent cohorts. Third, the progressive up-regulation of ribosome biogenesis pathways suggests a potential biological vulnerability, although functional and clinical validation remains necessary before these proteins can be used as prognostic or therapeutic markers.

Several limitations of this study should be acknowledged. First, the relatively small cohort, particularly the GC group, limits statistical power and generalizability of our findings. Second, the study compared different patients at different disease stages rather than following individual patients over time, which cannot capture the dynamic molecular changes within individual patients during disease progression. Third, the HpN-CG group, while *H. pylori* negative, represents patients with chronic gastritis rather than truly healthy individuals, which may affect the interpretation of *H. pylori*-specific effects. Fourth, the key findings were externally assessed using public RNA-seq databases, but RNA expression cannot substitute for direct protein-level validation, particularly for proteins such as those in Cluster 6 where RNA and protein patterns may diverge. Fifth, biopsy specimens from gastritis groups and surgical specimens from GC patients represent different tissue procurement methods that may introduce systematic bias. Sixth, the mechanistic roles of identified proteins, particularly the Cluster 4 ribosome-biogenesis module, require experimental confirmation through immunohistochemistry, immunoblotting, cell-line studies, and animal models. Seventh, our study did not include intestinal metaplasia or dysplasia stages, which are important intermediates in the Correa cascade. Future studies should address these limitations through larger, multi-center cohorts with longitudinal follow-up and functional validation experiments.

## Conclusion

Through integrated proteomic and bioinformatic analysis, we have systematically characterized the dynamic molecular changes occurring during *H. pylori*-associated gastric carcinogenesis across distinct pathological stages. Our study identified stage-specific protein expression patterns, including immune pathway activation upon *H. pylori* infection, progressive up-regulation of Cluster 4 ribosome-biogenesis proteins, and progressive down-regulation of oxidative phosphorylation proteins. These findings suggest candidate progression-associated protein modules that require further protein-level and functional validation. The concurrent decline in oxidative phosphorylation machinery underscores the importance of metabolic reprogramming in gastric carcinogenesis. These findings provide a molecular framework for understanding GC pathogenesis and highlight promising directions for future biomarker validation and therapeutic investigation.

## Supporting information

S1 Table Differentially expressed proteins across consecutive stages of Helicobacter pylori-associated gastric mucosal lesion progression. (XLSX)

S2 Table Mfuzz soft-clustering assignments and temporal protein-expression profiles across Helicobacter pylori-associated gastric mucosal lesion stages. (XLS)

S1 Fig External RNA-expression assessment of Cluster 6 hub genes in gastric cancer using GEPIA. (TIF)
