# Supplementary figures and images for: The quantitative proteomics analysis of multi-stage gastric mucosal lesions associated with Helicobacter pylori infection
Source: PLoS One. 2026 Jul 31;21(7):e0353347. doi: 10.1371/journal.pone.0353347 (PMC13426948; doi:10.1371/journal.pone.0353347)

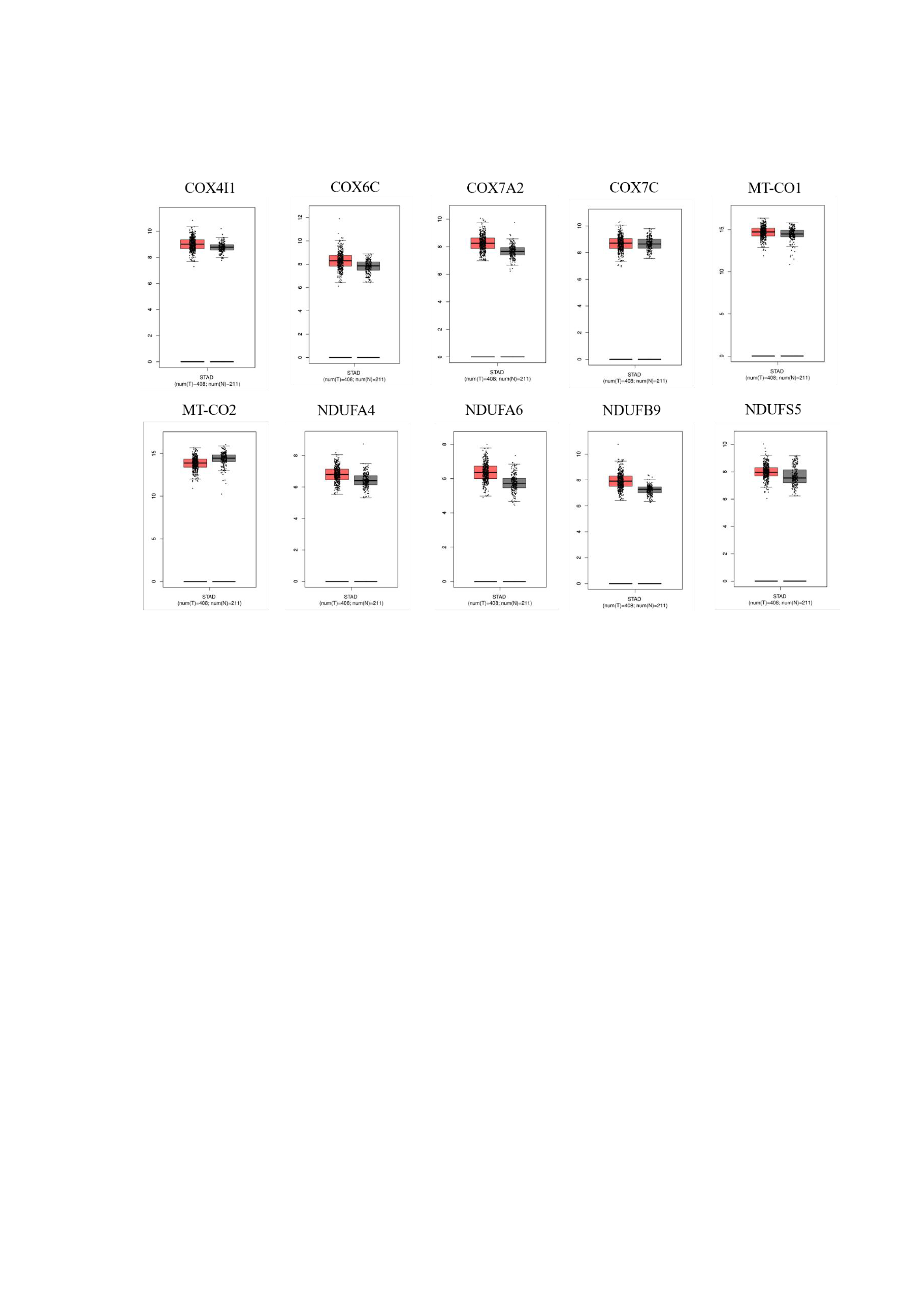

Supplement: S1 Fig — (TIF) [file pone.0353347.s003.tif]
